# SEOM clinical guidelines for cancer anorexia-cachexia syndrome (2023)

**DOI:** 10.1007/s12094-024-03502-8

**Published:** 2024-06-01

**Authors:** Ainara Soria Rivas, Yolanda Escobar Álvarez, Ana Blasco Cordellat, Margarita Majem Tarruella, Kevin Molina Mata, Marta Motilla de la Cámara, Mª del Mar Muñoz Sánchez, Marta Zafra Poves, Carmen Beato Zambrano, Luis Cabezón Gutierrez

**Affiliations:** 1 Medical Oncology Department, Hospital Univ. Ramón y Cajal, Madrid, Spain; 2 Medical Oncology Department, Hospital General Univ. Gregorio Marañón, Madrid, Spain; 3 Medical Oncology Department, Consorcio Hospital General Univ. de Valencia, Valencia, Spain; 4 Medical Oncology Department, Hospital de La Santa Creu I Sant Pau, Barcelona, Spain; 5 Medical Oncology Department, Hospital Duran I Reynals, Institut Català D’Oncologia L’Hospitalet (ICO), Barcelona, Spain; 6 Endocrinology and Nutrition Department, Hospital General Univ. Gregorio Marañón, Madrid, Spain; 7 Medical Oncology Department, Hospital General Virgen de La Luz, Cuenca, Spain; 8 Medical Oncology Department, Hospital Univ. Morales Meseguer, Murcia, Spain; 9 Medical Oncology Department, Hospital Univ. Virgen Macarena, Seville, Spain; 10 Medical Oncology Department, Hospital Univ. de Torrejón, Madrid, Spain

**Keywords:** Anorexia-cachexia syndrome, Malnutrition, Sarcopenia, Cancer, Anorexia, Cachexia

## Abstract

Cancer-related anorexia-cachexia syndrome (CACS) is a debilitating condition afflicting up to 80% of advanced-stage cancer patients. Characterized by progressive weight loss, muscle wasting, and metabolic abnormalities, CACS significantly compromises patients’ quality of life and treatment outcomes. This comprehensive review navigates through its intricate physiopathology, elucidating its stages and diagnostic methodologies. CACS manifests in three distinct stages: pre-cachexia, established cachexia, and refractory cachexia. Early detection is pivotal for effective intervention and is facilitated by screening tools, complemented by nutritional assessments and professional evaluations. The diagnostic process unravels the complex interplay of metabolic dysregulation and tumor-induced factors contributing to CACS. Management strategies, tailored to individual patient profiles, encompass a spectrum of nutritional interventions. These include dietary counseling, oral nutritional supplements, and, when necessary, enteral nutrition and a judicious use of parenteral nutrition. Specific recommendations for caloric intake, protein requirements, and essential nutrients address the unique challenges posed by CACS. While pharmacological agents like megestrol acetate may be considered, their use requires careful evaluation of potential risks. At its core, this review underscores the imperative for a holistic and personalized approach to managing CACS, integrating nutritional interventions and pharmacological strategies based on a nuanced understanding of patient’s condition.

## Introduction

Cancer-related anorexia-cachexia syndrome (CACS) comprises a multifactorial and debilitating condition prevalent among cancer patients. Characterized by a complex interplay of metabolic alterations, inflammation, and anorexia, CACS has a significant impact on the overall well-being of individuals undergoing cancer treatment [1].

CACS affects a substantial proportion of cancer patients, with prevalence rates varying across cancer types. The syndrome is most commonly observed in advanced stages of malignancy, affecting up to 80% of patients with advanced pancreatic cancer and approximately 60% of those with advanced lung cancer [2]. Its prevalence speaks to the clinical significance of CACS, emphasizing the need for a comprehensive understanding and effective management strategies.

Beyond its repercussions as a nutritional disorder, CACS profoundly affects treatment outcomes. The syndrome contributes to diminished treatment tolerance, impaired quality of life, and increased morbidity [3]. Cancer patients experiencing CACS are often challenged to adhere to therapeutic schedules, leading to compromised treatment efficacy and suboptimal disease control. Therefore, addressing the complexities of CACS is paramount if we are to optimize the overall success of oncologic interventions.

In conclusion, the intricate nature of CACS calls for a nuanced approach to its management in the oncological setting. Recognizing the prevalence of this syndrome and its implications for treatment outcomes underscores the imperative for continued research and the development of targeted interventions to enhance the overall care of cancer patients.

## Methodology

This guideline is based on a systematic review of relevant published studies and with the consensus of ten oncologists from SEOM (Spanish Society of Medical Oncology) who are experts in treatment, as well as an external review panel consisting of two experts designated by SEOM. The Infectious Diseases Society of America-US Public Health Service Grading System for Ranking Recommendations in Clinical Guidelines has been used to assign levels of evidence and grades of recommendation.

## Definition, incidence, and epidemiology

CACS is a complex metabolic syndrome associated with underlying illness and characterized by loss of muscle mass with or without loss of fat mass that occurs involuntarily. Anorexia, activation of inflammatory system, insulin resistance, and increased muscle protein breakdown are frequently associated with cachexia. Patients with CACS often have difficulty swallowing food and may experience nausea, vomiting, or an early feeling of satiety [1, 4, 5].

There are three stages of CACS: pre-cachexia, established cachexia, and refractory cachexia [6].

The hallmark of pre-cachexia is the presence of early clinical and metabolic signs such as anorexia and glucose intolerance that precede weight and muscle mass loss. The risk of progression to cachexia depends on the type of cancer, stage of the disease, the degree of systemic inflammation, food intake, and the response to antineoplastic therapy. Refractory cachexia is usually the result of a sustained state of cachexia or of rapidly progressing advanced cancer.

The incidence of CACS ranges from 20 to 40% in patients in the diagnostic phase and 70–80% in the advanced phase of the disease. The prevalence of anorexia-cachexia according to the primary tumor site is about 80% in pancreatic and gastric neoplasms; 54–60% in lung, prostate, and colon cancers, and 32–48% in breast cancers, sarcomas, lymphomas, and leukemias.

Moreover, cachexia is the direct cause of the patient’s death in more than 20% of these cases and it is estimated that death generally ensues when weight loss exceeds 30–40%. Nevertheless, approximately 50% of patients die *with* cachexia, but not of cachexia [7].

## Cancer anorexia-cachexia syndrome physiopathology

CACS physiopathology is based on a combination of metabolic and endocrinological mechanisms related to the tumor–host interaction. This interaction produces tumoral and humoral factors that include cytokines, neuropeptides, and hormones. The tumor can contribute to CACS by means of other causes, such as anatomical location or oncological treatment side effects.

### Metabolic and endocrinological factors

Tumor-related proinflammatory cytokine secretion is one of the main mechanisms underlying CACS, in particular interleukin-1 (IL-1), interleukin-6 (IL-6), tumor necrosis factor alpha (TNF-α), and C-reactive protein (CRP) [3]. All these cytokines have a catabolic and anorexigenic effect that causes weight and lean mass loss.

TNF-α was one of the first CACS endogen mediators to be discovered. It activates protein degradation through the proteasome-ubiquitin system, acting on different transcription factors, for instance, myoblast determination protein 1 and kappa B transcription nuclear factor (NF-kB). TNF-α decreases glucose and amino acid absorption by muscle tissue.

IL-1 induces an increase in corticotropin-releasing hormone, cholecystokinin, serotonin, and melanocortin levels, all of which are anorexigenic and decrease Y neuropeptide orexigenic agent production by the hypothalamus.

High IL-6 levels are associated with hypoalbuminemia, total protein decrease, lower body mass index, and anemia. In addition, elevated IL-6 levels correlate with a significantly shorter overall survival rate [8].

IL-1 and IL-6 reduce insulin production. Furthermore, cancer is assumed to be an alert state that increases glucose consumption due to glucagon, cortisol, and catecholamine release. This situation favors a negative metabolic balance in which catabolism predominates by means of organic nutrient degradation. Neoglycogenesis, an inefficient energy pathway, is activated and patients are in a prediabetic state which supports weight loss [9].

Leptin is a homeostatic protein produced by fatty tissue. When body weight decreases, leptin production is decreased with the consequent appetite stimulation by the central nervous system. In CACS patients, TNF-α and IL-1 are suspected to interfere with the leptin orexigenic response.

Likewise, a group of various transcription factors has been identified that plays an important role in CACS. The proteolysis-inducing factor (PIF) and lipid-mobilizing factor (LMF) destroy muscle mass and fatty deposits, releasing proteins and lipids into the bloodstream, thereby precipitating a diabetic state.

Adenosine triphosphate (ATP)-ubiquitin–proteasome pathway activation plays a crucial role in cancer-related proteolysis and proinflammatory cytokines stimulate ubiquitin messenger ARN production [7]. The ubiquitin–proteasome system, via NF-kB signaling, causes striated and cardiac muscle destruction. The effect of proinflammatory cytokines is directed against skeletal muscle myosin heavy chain [10].

The JAK/STAT pathway, which is active in a wide variety of solid tumors, is likewise involved in cancer-related sarcopenia [11].

In conclusion, although the most outstanding aspect of CACS is sarcopenia, fatty mass is also consumed.

### Tumor anatomical factors

Most gastrointestinal and head and neck tumors can cause mechanical nutritional issues. Dysphagia, abdominal pain, and/or stomach reflux can lead to a decline in food intake. Some tumors cause early satiety due to abdominal occupation, for instance, hepatomegaly, other organ infiltration, and ascites. Other neoplasms affect gastrointestinal motility as a result of intestinal or peritoneal infiltration and nutritional malabsorption may occur owing to pancreatic tumor involvement.

### Cancer treatment factors

Oncological treatments can also impact sarcopenia.

Surgery may lead to malnutrition as a result of increased nutritional needs. Swallowing, malabsorption, and/or digestive surgery-related changes can provoke malnutrition.

Radiotherapy and/or chemotherapy can trigger oral mucositis, xerostomia, dysgeusia, appetite loss, nausea, vomiting, and enteritis. These potential side effects make food intake difficult, lower caloric input, and exacerbate nutrient loss.

Finally, specific cancer treatments such as anti-androgens for prostate cancer or different types of antiangiogenics may cause muscle loss [12, 13].

## CACS diagnosis, screening, and evaluation methods

### ***Characteristics of CACS stages: ***[1, 6]

- Pre-cachexia: systemic inflammation with weight loss of <5% of the patient’s body weight.
- Cachexia: systemic inflammation with weight loss >5% or BMI < 20 kg/m^2^ and weight loss >2% or sarcopenia with weight loss >2%. In this stage, there is reduced food intake and systemic inflammation. Nutritional intervention at this stage is critical.
- Refractory cachexia: characterized by irreversible catabolism, resulting in poor functional status. Nutritional intervention at this point will be aimed at preventing exacerbation, taking into account the patient’s confort.

### Diagnosis

The diagnosis of CACS is based on regular systematic nutritional evaluation of all cancer patients, both at the time of diagnosis and during treatment. It is considered indicated when life expectancy exceeds 3–6 months. In individuals with an expected survival of less than a few weeks, screening for eating-related distress should be carried out in both the patient and family members [14]. Such assessments should be quarterly or earlier when treatment cases make nutritional modifications foreseeable.

Screening methods enable early diagnosis and intervention. They should be simple, fast, and have adequate sensitivity and specificity. Estimated nutritional intake, weight changes, and BMI should be assessed regularly (at the time of diagnosis and when therapeutic changes are made) [15, 16].

Several nutritional screening tools are available (Table 1):

- The Nutrition Risk Screening (NRS) 2002 was created for hospitalized patients and examines three components: impaired nutritional status, severity of disease, and age [17].
- The Malnutrition Universal Screening Tool (MUST) has demonstrated its robustness for use in adult patients across all healthcare settings including oncology. It is based on BMI, percentage of weight loss, and the effect of acute disease [18].
- The Malnutrition Screening Tool (MST) has been validated as a predictor of malnutrition risk in outpatient cancer patients undergoing treatment and consists of two questions regarding recent, unintentional weight loss and diminished appetite affecting dietary intake [19].
- The Patient-Generated Subjective Global Assessment (GP-VGS) has been validated in transplant, HIV, surgical, and oncology patients. It is based on weight loss, type of disease, degree of metabolic stress, physical examination, and patient self-assessment. It established three main degrees: adequate nutritional status, risk of malnutrition or moderate malnutrition, and severe malnutrition [20].
- Nutriscore® was specifically designed for oncology patients, validated in the outpatient setting, although it is also useful in hospitalized patients. It is based on weight loss, decrease in appetite, tumor type, and treatment. Subjects are classified into two groups (nutritional risk or no risk). This tool has been validated in the Spanish population [21].

**Table 1:** Nutritional risk assessment tools

	NRS-2002 [17]	MUST [18]	MST [19]	VGS [20]	Nutriscore® [21]
Parameters	BMI, weight loss, reduced dietary intake, ICU patient	BMI, unplanned weight loss, no nutritional intake for >5 days	Weight loss, reduced dietary intake	Clinical report (weight loss, symptoms, reduced dietary intake, and functional status) + physical exam	Weight loss, appetite decrease, tumor type, and treatment
Type of patients	Hospitalized patients	Outpatients and inpatients	Hospitalized patients and oncological outpatients in treatment	HIV, transplant, surgical, and oncology outpatients	Oncology outpatients

There is no consensus as to how to perform screening and what cut-off points are required to undertake assessment [22].

Finally, in those patients with alterations detected on screening, an objective, quantitative diagnostic evaluation of nutritional status should be performed by nutrition professional.

Therefore, the clinical and dietary history must be evaluated, including a physical and anthropometric examination, analytical determinations, and assessment of the disease and symptoms, including the capacity for exercise and the ability of the patient to eat [23].

### Specific evaluation methods

1. Anthropometric parameters: BMI, percentage of weight loss (more than 10% in the last 6 months or 5% in the last 3 months), brachial circumference (<20 cm or >2 cm decrease on two determinations), muscle mass assessment
2. Biochemical parameters: systemic inflammation data such as albumin, prealbumin, serum C-reactive protein.
3. Clinical parameters: digestive symptoms associated with cancer and treatment with affecting nutrition (anorexia, nausea, taste alterations, smell, stomatitis, constipation, diarrhea, dysphagia, pain).
4. Dietetic parameters: type of food intake (percentage of protein, caloric)
5. Functional status: ECOG, asthenia, physical activity, dyspnea, psychosocial distress.

## Nutritional approach to advanced cancer: nutritional counseling, oral supplements, enteral nutrition, and parenteral nutrition

### Overview

**Fig. 1:**
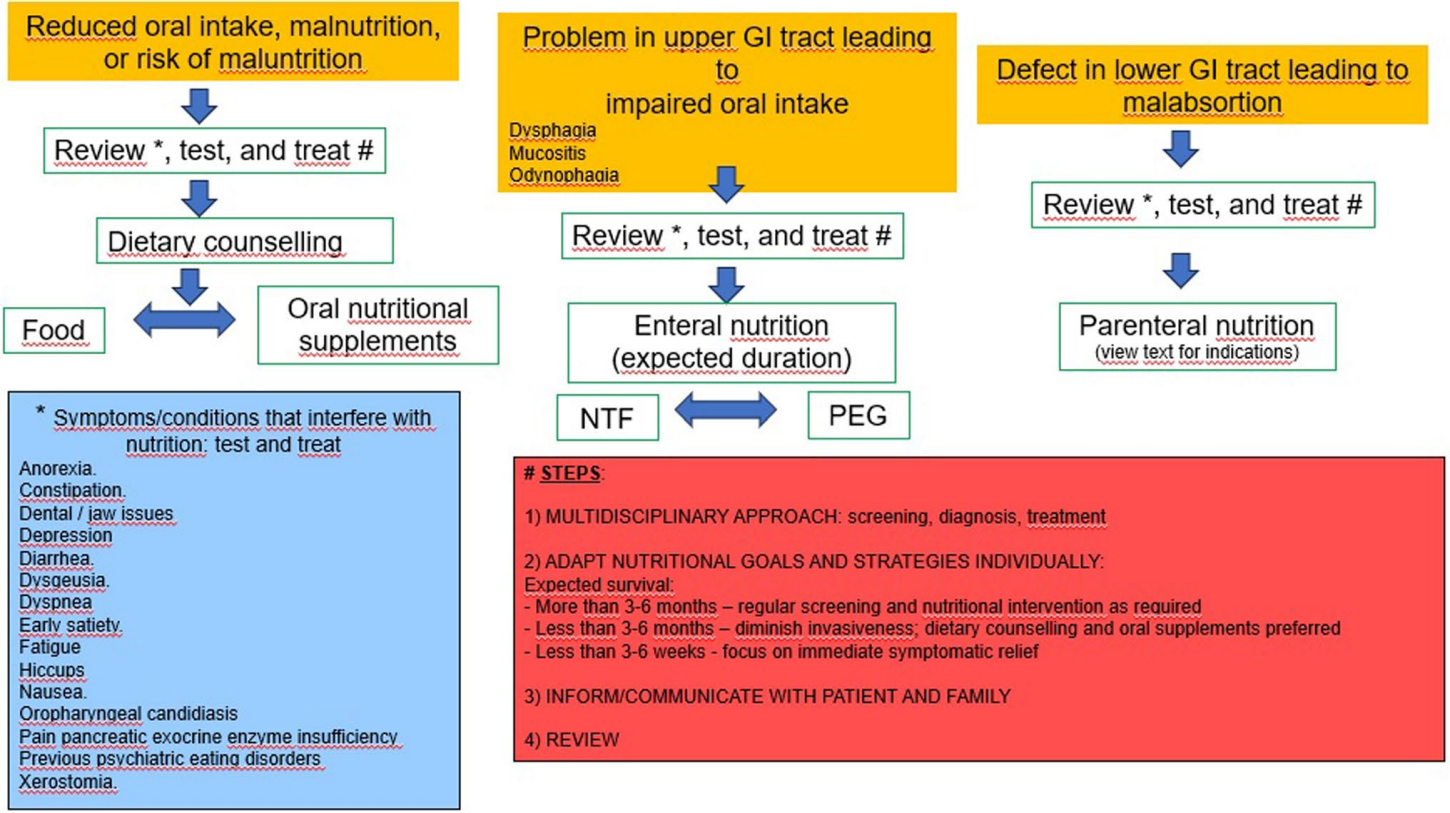
Nutritional counseling, oral supplements, enteral nutrition, and parenteral nutrition

The degree of invasiveness of the nutritional intervention should be individualized, based on the person’s nutritional status, cancer type and stage, comorbidities, overall medical treatment plan, and gastrointestinal (GI) tract functioning. There are several symptoms and conditions that should also be checked. The perspective of nutritional care should change in parallel to the cancer evolution. During active anticancer treatment, patients might be offered different nutritional treatment options, if required. At the end of life, care should focus on symptom management [14–16, 24] (Fig. 1).

### Dietary counseling (DC) and oral nutritional supplements (ONS)

Nutritional support in patients able to eat should be based on dietary counseling (DC), guidance on choosing high-energy, high-protein foods, enriching their diet, and using oral nutritional supplements (ONS).

ONSs are a balanced mixture of macronutrients and micronutrients available as liquid feeds, puddings, and powdered formulations reconstituted with milk or water. They are available in a range of presentations, flavors, and formulations, including those that are high in fiber or that contain milk, as well as juices, and yoghurt-like products.

Two systematic reviews have proven that DC is generally effective in increasing dietary intake, body weight, and quality of life in subjects undergoing radiotherapy, with some suggestion that dietary counseling may also improve symptoms affected by nutrition, complications, response to anticancer treatment, and survival [25, 26]. A third systematic review added that providing standard ONS without dietary counseling was not effective [27]. On the other hand, a meta-analysis of individuals receiving chemotherapy revealed positive effects of DC with or without ONS [28].

Finally, several randomized trials have been published regarding the effects of N-3 fatty acids in cancer-related cachexia. Overall, studies were heterogeneous and inadequately powered to demonstrate effects on treatment toxicity or survival. No negative effects of ONS were reported. Most trials suggested that N3P-ONS benefitted weight, lean body mass, and some aspects of QoL when given to patients on active treatment [29, 30].

### Enteral nutrition (EN)

Patients with head and neck (HNSCC) or upper GI cancers are at exceptional risk of malnutrition that may be caused by dysphagia due to obstruction, motility dysfunction, or severe mucositis induced by anticancer treatment. Nutritional status can be maintained or enhanced by EN in these patients in whom oral intake or food transportation is impaired [25]. Several RCTs have determined that EN and parenteral nutrition have similar efficacy and complication rates. Given that EN is more physiological, simpler, and less expensive, it is preferred if small bowel function is preserved [26].

Several RCTs have compared nose tube (NTF) and percutaneous endoscopic gastrostomy (PEG) in head and neck cancer patients: both achieved similar nutritional results over the long term. Meta-analyses have failed to detect any significant differences with respect to overall complication rates between the two [26].

### Parenteral nutrition (PN)

PN did not significantly improve overall survival in advanced cancer patients in one systematic review, but did increase complication rates [31]. Nevertheless, for some subjects on antineoplastic therapy, PN may be appropriate when their cancer or treatment affects their ability to ingest or absorb nutrients and in cases in which life expectancy is months-years [32]. PN might be considered when their prognosis exceeds 2 months, as well as when their mental and physical status and social support are sufficient to improve QoL. Key indicators for benefit are ECOG PS 0–2, low systemic inflammation (normal levels of serum albumin, modified Glasgow Prognostic Score <2), and non-metastatic TNM stage [14]. The pros (possible physical, psychological benefits) and cons (complications, futility, inequality) of PN should be reviewed individually in a multidisciplinary scenario. The risks of PN outweigh benefits for patients with a prognosis of fewer than 2 months [24]. Discontinuation of PN near the end of life is appropriate [15].

## Caloric intake and specific nutrient requirements

The goal of nutritional treatment is to stabilize or increase caloric-protein intake to meet oral nutritional requirements, reduce inflammation, optimize the person’s nutritional status, and prevent deleterious effects related to malnutrition [16].

### Caloric requirements

(TEE) includes three core components: resting energy expenditure (REE); the thermic effect of food (TEF), and physical activity. In oncology patients, energy expenditure can vary considerably based on factors such as inflammation, body composition, amount of brown adipose tissue, level of physical activity, tumor type, and tumor size [33]. For patients with CACS, key determinants of energy expenditure include advanced age, tumor progression, comorbidities, nutritional deficiencies, and certain medications or medical interventions.

REE, assessed using indirect calorimetry (gold standard), exhibits tremendous variability in these patients and can be increased (50% of CACS patients), normal, or even decreased compared to healthy individuals. While REE tends to be elevated in cancer patients, when TEE is calculated, it is similar to the values predicted for healthy individuals, likely due to lower daily physical activity in CACS patients [33, 34].

A theoretical model proposes that the hypoxic tumor microenvironment induces an energy deficit, particularly during fasting, by means of a vicious cycle of energy loss [33]. Muscle wasting in cachexia arises from a combination of reduced protein synthesis and increased protein catabolism. Lipolysis is triggered by enhanced activation of the central nervous system and by cytokines such as IL-1, IL-6, TNF-α, and TNF-γ, as well as zinc glycoprotein-α [35].

### Protein requirements

There is no evidence that clarifies protein requirements in patients with CACS. Small randomized clinical trials suggest that increased protein intake results in an improved protein balance and enhanced intramuscular protein synthesis. Decreased anabolic factors and increased protein catabolic factors have been observed in CACS patients. Overexpression of uncoupling proteins at the mitochondrial level has also been documented in situations of cachexia, which promotes thermogenesis and, consequently, energy consumption [36, 37].

### Specific nutritional requirements

1. Vitamins and minerals: In a systematic review, the authors concluded that there is insufficient evidence to recommend intervention, including the use of magnesium; vitamin E + omega-3 fatty acids; vitamin D; vitamin C; beta-hydroxy-b-methylbutyrate (HMB) + arginine + glutamine, and L-carnitine [16, 38].
2. Amino acids (aa): In a 2023 review of supplementation with branched-chain amino acids in individuals undergoing oncology treatment, two studies were found with disparate results (decreased malnutrition during or post-chemotherapy in one, while in the other, a possible increase in tumor growth) [39]. Therefore, there is not enough evidence regarding a supplementation. In a meta-analysis published in 2022 of the use of HMB, the authors found evidence of improved muscle mass and function, without any related adverse event [38]. There is not enough evidence to recommend glutamine to improve nutritional status in patients with CACS, unless it is part of the HMB/arginine/glutamine combination [40].
3. Immunonutrition: According to a 2012 meta-analysis, oral or enteral administration of these types of immunomodulatory formulas was associated with a decrease in infectious complications and shorter hospital stay in the perioperative period [41]. Nevertheless, there is no evidence to support their use in CACS patients*.*
4. Omega-3 fatty acids: The authors of two meta-analyses from 2015 and 2018 have suggested that omega-3 fatty acid use is safe, well-tolerated, and may improve weight, lean mass, and survival. Nonetheless, the clinical trials on which these findings are based are relatively small and heterogeneous [42, 43]. Therefore, the data available regarding the use of omega-3 fatty acids are not robust enough as to recommend their use in all patients with cachexia.

## Pharmacological treatment of anorexia

The evidence is insufficient to firmly support any drug to treat cancer-associated anorexia/cachexia syndrome (CACS), and its use depends on the values and preferences of individual patients and other considerations, such as degree of anorexia or weight loss, comorbidities, risk of adverse effects, life expectancy, and goals of care. The main benefits purportedly associated with these drugs are increased appetite and moderate weight gain, albeit without improving survival.

Various drugs have been studied in the context of CACS, although only two available options have demonstrated any benefit (progesterone analogues and glucocorticoids). All are summarized in Table 2.

**Table 2:** Pharmacological interventions for the treatment of CACS in patients with cancer

Drug	Strength of recommendation	Strength of the evidence	Effects
Glucocorticoids [44, 45]	Moderate in favor	Intermediate	Increased appetite and sense of well-being
Progesterone analogs [44, 46–48]	Moderate in favor	Intermediate	Weight gain; increased appetite
Androgens [49]	No recommendation	Low	Improved LBM and QoL
Anamorelin [50]	No recommendation	Intermediate	Increased body weight and LBM
Cannabinoids [51]	Weak against	Low	Increased appetite
Cyproheptadine [52]	No recommendation	Low	Increased appetite
Hydrazine sulfate [53]	Strong against	Intermediate	No effects
Melatonin [54]	Weak against	Low	Decreased serum TNF concentrations
NSAIDs [55]	No recommendation	Low	Prevention of weight loss; improved QoL
Olanzapine [56]	No recommendation	Low	Increased appetite
Thalidomide [57]	No recommendation	Low	Reduced cytokines levels; improved QoL
TNF inhibitors [58]	Moderate against	Intermediate	Inhibition of TNF-alpha production

*LBM* lean body mass, *QoL* quality of life, *NSAIDs* non-steroidal anti-inflammatory drugs, *TNF* tumor necrosis factor

**Glucocorticoids**: For a time, glucocorticoids were first-line therapy to stimulate CACS patients’ appetite. Glucocorticoids improve appetite to a similar degree as that seen with progesterone analogs. Nevertheless, given the toxicities and decline in efficacy associated with long-term use, their role as an appetite stimulant is often limited to those with an estimated life expectancy of weeks to a few months. Corticosteroids is a class of drugs that encompasses several agents having variable glucocorticoid, mineralocorticoid, and anti-inflammatory potency. Dexamethasone, prednisolone, and methylprednisolone are used most frequently. Several randomized controlled trials (RCTs) investigating the effects of corticosteroids on appetite in patients with advanced cancer have been published [44, 45]. Many subjects treated with glucocorticoids experience increased in appetite and sense of well-being though not weight gain, in comparison against placebo. However, the anti-anorexic effect of corticosteroids is temporary and often disappears after a few weeks. Furthermore, prolonged steroid therapy produces myopathy and a wide assortment of other side effects. A reasonable dose of dexamethasone in this setting is 4 mg/day, although lower doses may also be effective [15].

**Progesterone analogs**: Megestrol acetate (MA) improves appetite and body weight in patients with cancer-related cachexia, but the weight gained is primarily in the form of adipose tissue rather than skeletal muscle [44, 46]. Toxicities include thromboembolic events, edema, and adrenal suppression. Similar findings have been observed with medroxyprogesterone acetate (MPA) [47].

A number of prospective, controlled randomized trials, as well as several systematic reviews and meta-analyses have confirmed the modest efficacy of MA and MPA to palliate CACS. A 2013 Cochrane review of MA for CACS [44] concluded that when compared to placebo, MA significantly improves appetite (relative risk 2.57) and weight gain (relative risk 1.55). However, no consistent improvement in quality of life (QoL) was observed and no data on muscle mass or physical function were reported. In the trials analyzed, MA was used in doses of 160–800 mg/day and improvement in weight appeared to be greater at doses >160 mg/day, while no dose effect was observed for appetite. There was a positive correlation between appetite stimulation and increasing MA doses ranging from 160 to 800 mg/day. A higher dose, 1280 mg/day, proved not to be more effective [48].

Edema, thromboembolic events, and deaths occurred more in patients treated with MA. The authors concluded that MA was associated with increased mortality (relative risk 1.42, 95% CI 1.04–1.94), with greater risk at doses of ≥800 mg/day, although a subsequent update concluded that such detrimental effects on survival are nonexistent [46, 48].

**Other treatments**: The evidence is inconclusive regarding the benefits of many other treatments for CACS including olanzapine; androgens, and selective androgen receptor modulators; anamorelin; cyproheptadine; long-chain omega-3 fatty acids, vitamins, minerals, and other dietary supplements; non-steroidal anti-inflammatory drugs (NSAIDs); thalidomide; mirtazapine; hydrazine sulfate; TNF inhibitors; melatonin; insulin, and combination therapies [14–16, 49–58]. None of these approaches can be recommended at present.

## Recommendation summary

- For patients with HNSCC or upper GI cancers, especially those undergoing CT/RT, thorough evaluation and monitoring nutritional status is encouraged [I, A].
- Standard nutritional risk screening with a validated tool performed at regular intervals is recommended in all cancer patients under treatment with life expectancy >3 to 6 months (V, B).
- When necessary, nutritional support is recommended in all patients receiving anticancer treatment with an expected survival of more than a few months [V, B].
- In patients with expected survival of less than a few weeks, comfort-directed care is recommended [V, B].
- DC should be the first step of nutritional support offered to cachectic or at-risk patients who are able to eat [II, B].
- ONS can be supplied as part of DC to improve energy intake and induce weight gain [II, B].
- EN to maintain nutritional status is recommended if oral feeding is expected to be insufficient for more than a few days [I, A].
- If EN is predicted to be required for more than 1 month, PEG rather than NTF is recommended [II, C].
- PN, managed by a multidisciplinary team, might be used in oncologic patients if their survival is expected to be severely compromised by progressive malnutrition and not by the cancer itself [V, B].
- Total energy expenditure (TEE) should be 25–30 kcal/kg day, adapting it to the evolution of nutritional status [V, B].
- Protein intake is recommended to be 1–2 g/kg day [V, B].
- Vitamins and minerals should be supplied similarly to the recommended daily amount. The use of high doses of micronutrients in the absence of specific deficiencies is discouraged [I, A].
- There is insufficient evidence to warrant recommendation of branched-chain amino acids (aa) or other aa or their metabolites to improve lean mass [II, C]. Supplementation with HMB is recommended to improve muscle mass and function [II, B]. The use of glutamine is optional in patients with CACS [II, C].
- Immunonutrition may be used in the perioperative period of an upper GI cancer patient undergoing surgery, but there is not enough evidence for CACS [I, A].
- Corticosteroids may be used to increase appetite in patients with an estimated life expectancy of weeks to a few months (I, B).
- The progesterone analog, megestrol acetate, may be used to increase appetite and weight gain in CACS (I, B).

## References

[1] Fearon K, Strasser F, Anker SD, Bosaeus I, Bruera E, Fainsinger RL, et al. Definition and classification of cancer cachexia: an international consensus. Lancet Oncol. 2011;12(5):489–95. 10.1016/S1470-2045(10)70218-7. 21296615 10.1016/S1470-2045(10)70218-7

[2] Argilés JM, Busquets S, Stemmler B, López-Soriano FJ. Cancer cachexia: understanding the molecular basis. Nat Rev Cancer. 2014;14(11):754–62. 10.1038/nrc3829. 25291291 10.1038/nrc3829

[3] Tisdale MJ. Cachexia in cancer patients. Nat Rev Cancer. 2002;2(11):862–71. 10.1038/nrc927. 12415256 10.1038/nrc927

[4] Dev R. Measuring cachexia-diagnostic criteria. Ann Palliat Med. 2019;8(1):24–32. 10.21037/apm.2018.08.07. 30525765 10.21037/apm.2018.08.07

[5] Tuca A, Jimenez-Fonseca P, Gascón P. Clinical evaluation and optimal management of cancer cachexia. Crit Rev Oncol Hematol. 2013;88(3):625–36. 10.1016/j.critrevonc.2013.07.015. 23953794 10.1016/j.critrevonc.2013.07.015

[6] Wiegert EVM, de Oliveira LC, Calixto-Lima L, Chaves GV, Silva Lopes MS, Peres WAF. New cancer cachexia staging system for use in clinical practice. Nutrition. 2021;90:111271. 10.1016/j.nut.2021.111271. 34004417 10.1016/j.nut.2021.111271

[7] Llovera M, García-Martínez C, Agell N, F J López-Soriano FJ, J M Argilés JM. TNF can directly induce the expression of ubiquitin-dependent proteolytic system in rat soleus muscles. Biochem Biophys Res Commun. 1997;230(2):238–41. 10.1006/bbrc.1996.5827. 10.1006/bbrc.1996.5827 9016756

[8] Kuroda K, Nakashima J, Kanao K, Kikuchi E, Miyajima A, Horiguchi Y, et al. Interleukin 6 is associated with cachexia in patients with prostate cancer. Urology. 2007;69(1):113–7. 10.1016/j.urology.2006.09.039. 17270630 10.1016/j.urology.2006.09.039

[9] Staal-van den Brekel AJ, Dentener MA, Schols AM, Buurman WA, Wouters EF. Increased resting energy expenditure and weight loss are related to a systemic inflammatory response in lung cancer patients. J Clin Oncol. 1995;13(10):2600–5. 10.1200/JCO.1995.13.10.2600. 10.1200/JCO.1995.13.10.2600 7595713

[10] Acharyya S, Ladner KJ, Nelsen LL, Damrauer J, Reiser PJ, Swoap S, et al. Cancer cachexia is regulated by selective targeting of skeletal muscle gene products. J Clin Invest. 2004;114(3):370–8. 10.1172/JCI20174. 15286803 10.1172/JCI20174 PMC484974

[11] Quintás-Cardama A, Verstovsek S. Molecular pathways: Jak/STAT pathway: mutations, inhibitors, and resistance. Clin Cancer Res. 2013;19(8):1933–40. 10.1158/1078-0432.CCR-12-0284. 10.1158/1078-0432.CCR-12-0284 PMC5021219 23406773

[12] Chiang PK, Tsai WK, Chiu AW, Lin JB, Yang FY, Lee J. Muscle loss during androgen deprivation therapy is associated with higher risk of non-cancer mortality in high-risk prostate cancer. Front Oncol. 2021;11:722652. 10.3389/fonc.2021.722652. 34604058 10.3389/fonc.2021.722652 PMC8485032

[13] Colomba E, Alves Costa Silva C, Le Teuff G, Elmawieh J, Afonso D, Benchimol-Zouari A, et al. Weight and skeletal muscle loss with cabozantinib in metastatic renal cell carcinoma. J Cachexia Sarcopenia Muscle. 2022;13(5):2405–2416. 10.1002/jcsm.13021. 10.1002/jcsm.13021 PMC9530538 35903892

[14] Arends J, Strasser F, Gonella S, Solheim TS, Madeddu C, Ravasco P, et al. ESMO Guidelines Committee. Electronic address: clinicalguidelines@esmo.org. Cancer cachexia in adult patients: ESMO Clinical Practice Guidelines. ESMO Open. 2021;6(3):100092. 10.1016/j.esmoop.2021.100092 10.1016/j.esmoop.2021.100092 PMC8233663 34144781

[15] Roeland EJ, Bohlke K, Baracos VE, Bruera E, Del Fabbro E, Dixon S, et al. Management of cancer cachexia: ASCO guideline. J Clin Oncol. 2020;38(21):2438–53. 10.1200/JCO.20.00611. 32432946 10.1200/JCO.20.00611

[16] Muscaritoli M, Arends J, Bachmann P, Baracos V, Barthelemy N, Bertz H, et al. ESPEN practical guideline: clinical nutrition in cancer. Clin Nutr. 2021;40(5):2898–913. 10.1016/j.clnu.2021.02.005. 33946039 10.1016/j.clnu.2021.02.005

[17] Kondrup J, Rasmussen HH, Hamberg O, Stanga Z; Ad Hoc ESPEN Working Group. Nutritional risk screening (NRS 2002): a new method based on an analysis of controlled clinical trials. Clin Nutr. 2003;22(3):321–36. 10.1016/s0261-5614(02)00214-5 10.1016/s0261-5614(02)00214-5 12765673

[18] Stratton RJ, Hackston A, Longmore D, Dixon R, Price S, Stroud M, et al. Malnutrition in hospital outpatients and inpatients: prevalence, concurrent validity and ease of use of the ‘malnutrition universal screening tool’ (‘MUST’) for adults. Br J Nutr. 2004;92(5):799–808. 10.1079/bjn20041258. 15533269 10.1079/bjn20041258

[19] Isenring E, Cross G, Daniels L, Kellett E, Koczwara B. Validity of the malnutrition screening tool as an effective predictor of nutritional risk in oncology outpatients receiving chemotherapy. Support Care Cancer. 2006;14(11):1152–6. 10.1007/s00520-006-0070-5. 16622648 10.1007/s00520-006-0070-5

[20] Abbott J, Teleni L, McKavanagh D, Watson J, McCarthy AL, Isenring E. Patient-Generated subjective global assessment short form (PG-SGA SF) is a valid screening tool in chemotherapy outpatients. Support Care Cancer. 2016;24(9):3883–7. 10.1007/s00520-016-3196-0. 27095352 10.1007/s00520-016-3196-0

[21] Arribas L, Hurtós L, Sendrós MJ, Peiró I, Salleras N, Fort E, et al. NUTRISCORE: a new nutritional screening tool for oncological outpatients. Nutrition. 2017;33:297–303. 10.1016/j.nut.2016.07.015. 27751743 10.1016/j.nut.2016.07.015

[22] Escobar Y, Ramchandani A, Salgado M, Castillo-Trujillo A, Martínez de Castro E, Diaz de Corcuera I, et al. What do patients and oncologists think about the evaluation and management of cancer-related anorexia-cachexia? The Quasar_SEOM study. Clin Transl Oncol 2023;25:3479–91. 10.1007/s12094-023-03212-7 10.1007/s12094-023-03212-7 37289352

[23] Virizuela JA, Camblor-Álvarez M, Luengo-Pérez LM, Grande E, Álvarez-Hernández J, Sendrós-Madroño MJ, et al. Nutritional support and parenteral nutrition in cancer patients: an expert consensus report. Clin Transl Oncol. 2018;20(5):619–29. 10.1007/s12094-017-1757-4. 29043569 10.1007/s12094-017-1757-4

[24] Alderman B, Lindsey A, Amano K, Bouleuc C, Davis M, Lister-Flynn S, et al. Multinational Association of Supportive Care in Cancer (MASCC) expert opinion/guidance on the use of clinically assisted nutrition in patients. Advanced cancer. Supportive Care in Cancer. 2022;30(4):2983–92. 10.1007/s00520-021-06613-y. 10.1007/s00520-021-06613-y PMC8857106 34665311

[25] Garg S, Yoo J, Winquist E. Nutritional support for head and neck & cancer patients receiving radiotherapy: a systematic review. Support Care Cancer. 2010;18(6):667–77.44. 10.1007/s00520-009-0686-3. 10.1007/s00520-009-0686-3 19582484

[26] Langius JA, Zandbergen MC, Eerenstein SE, et al. Effect of nutritional interventions on nutritional status, quality of life and mortality inpatients with head and neck cancer receiving (chemo)radiotherapy: a systematic review. Clin Nutr. 2013;32(5):671–8. 10.1016/j.clnu.2013.06.012. 23845384 10.1016/j.clnu.2013.06.012

[27] JLC, Leong LP, Lim SL. Nutrition intervention approaches to reduce malnutrition in oncology patients: a systematic review. Support Care Cancer. 2016;24(1):469–80. 10.1007/s00520-015-2958-4. 10.1007/s00520-015-2958-4 26404858

[28] Van der Schueren MAE, Laviano A, Blanchard H, Jourdan M, Arends J, Baracos VE. Systematic review and meta-analysis of the evidence for oral nutritional intervention on nutritional and clinical outcomes during chemo(radio)therapy: current evidence and guidance for design of future trials. Ann Oncol. 2018;29(5):1141–53. 10.1093/annonc/mdy114. 29788170 10.1093/annonc/mdy114 PMC5961292

[29] Van der Meij BS, van Bokhorst-de van der Schueren MA, Langius JA, Brouwer IA, van Leeuwen PAM. N-3 PUFAs in cancer, surgery, and critical care: a systematic review on clinical effects, incorporation, and washout of oral or enteral compared with parenteral supplementation. Am J Clin Nutr. 2011;94(5):1248–65. 10.3945/ajcn.110.007377. 10.3945/ajcn.110.007377 21940600

[30] Dewey A, Baughan C, Dean T, Higgins B, Johnson I. Eicosapentaenoic acid (EPA, anomega-3 fatty acid fromfish oils) for the treatment of cancer cachexia. Cochrane Database Syst Rev. 2007;1:CD004597. 10.1002/14651858.CD004597.pub2. 10.1002/14651858.CD004597.pub2 PMC6464930 17253515

[31] Chow R, Bruera E, Arends J, Walsh D, Strasser F, Isenring E, et al. Enteral and parenteral nutrition in cancer, a comparison of complication rates: an updated systematic review and (cumulative) meta-analysis. Support Care Cancer. 2020;28(3):979–1010. 10.1007/s00520-019-05145-w. 31813021 10.1007/s00520-019-05145-w

[32] Cotogni P. Enteral versus parenteral nutrition in cancer patients: evidences and controversies. Ann Palliat Med. 2016;5(1):42–9. 10.3978/j.issn.2224-5820.2016.01.05. 26841814 10.3978/j.issn.2224-5820.2016.01.05

[33] Purcell SA, Elliott SA, Baracos VE, Chu QS, Prado CM. Key determinants of energy expenditure in cancer and implications for clinical practice. Eur J Clin Nutr. 2016;70(11):1230–8. 10.1038/ejcn.2016.96. 27273068 10.1038/ejcn.2016.96

[34] Gangadharan A, Choi SE, Hassan A, Ayoub NM, Durante G, Balwani S, et al. Protein calorie malnutrition, nutritional intervention and personalized cancer care. Oncotarget. 2017;8(14):24009–30. 10.18632/oncotarget.15103. 28177923 10.18632/oncotarget.15103 PMC5410360

[35] Hegde M, Daimary UD, Girisa S, Kumar A, Kunnumakkara AB. Tumor cell anabolism and host tissue catabolism-energetic inefficiency during cancer cachexia. Exp Biol Med (Maywood). 2022;247(9):713–33. 10.1177/15353702221087962. 35521962 10.1177/15353702221087962 PMC9134760

[36] Deutz NE, Safar A, Schutzler S, Memelink R, Ferrando A, Spencer H, et al. Muscle protein synthesis in cancer patients can be stimulated with a specially formulated medical food. Clin Nutr (Edinb). 2011;30(6):759–68. 10.1016/j.clnu.2011.05.008. 10.1016/j.clnu.2011.05.008 PMC3964623 21683485

[37] Hunter DC, Weintraub M, Blackburn GL, Bistrian BR. Branched chain amino acids as the protein component of parenteral nutrition in cancer cachexia. Br J Surg. 1989;76(2):149–53. 10.1002/bjs.1800760215. 2495147 10.1002/bjs.1800760215

[38] Prado CM, Orsso CE, Pereira SL, Atherton PJ, Deutz NEP. Effects of β-hydroxy β-methylbutyrate (HMB) supplementation on muscle mass, function, and other outcomes in patients with cancer: a systematic review. J Cachexia Sarcopenia Muscle. 2022;13(3):1623–41. 10.1002/jcsm.12952. 35301826 10.1002/jcsm.12952 PMC9178154

[39] Lee K, Blanton C. The effect of branched-chain amino acid supplementation on cancer treatment. Nutr Health. 2023;29(4):621–35. 10.1177/02601060231153428. 36703299 10.1177/02601060231153428

[40] Prado CM, Purcell SA, Laviano A. Nutrition interventions to treat low muscle mass in cancer. J Cachexia Sarcopenia Muscle. 2020;11(2):366–80. 10.1002/jcsm.12525. 31916411 10.1002/jcsm.12525 PMC7113510

[41] Marimuthu K, Varadhan KK, Ljungqvist O, Lobo DN. A meta-analysis of the effect of combinations of immune modulating nutrients on outcome in patients undergoing major open gastrointestinal surgery. Ann Surg. 2012;255(6):1060–8. 10.1097/SLA.0b013e318252edf8. 22549749 10.1097/SLA.0b013e318252edf8

[42] de Aguiar Pastore Silva J, Emilia de Souza Fabre M, Waitzberg DL. Omega-3 supplements for patients in chemotherapy and/or radiotherapy: a systematic review. Clin Nutr (Edinb) 2015;34(3):359–66. 10.1016/j.clnu.2014.11.005. 10.1016/j.clnu.2014.11.005 25907586

[43] Sanchez-Lara K, Turcott JG, Juarez-Hernandez E, Nuñez-Valencia C, Villanueva G, Guevara P, et al. Effects of an oral nutritional supplement containing eicosapentaenoic acid on nutritional and clinical outcomes in patients with advanced non-small cell lung cancer: randomised trial. Clin Nutr(Edinb) 2014;33(6):1017–23. 10.1016/j.clnu.2014.03.006. 10.1016/j.clnu.2014.03.006 24746976

[44] Currow DC, Glare P, Louw S, Martin P, Clark K, Fazekas B, et al. A randomised, double blind, placebo-controlled trial of megestrol acetate or dexamethasone in treating symptomatic anorexia in people with advanced cancer. Sci Rep. 2021;11(1):2421. 10.1038/s41598-021-82120-8. 33510313 10.1038/s41598-021-82120-8 PMC7844230

[45] Willox JC, Corr J, Shaw J, Richardson M, Calman KC, Drennan M. Prednisolone as an appetite stimulant in patients with cancer. Br Med J (Clin Res Ed). 1984;288(6410):27. 10.1136/bmj.288.6410.27. 10.1136/bmj.288.6410.27 PMC1444189 6418303

[46] Ruiz Garcia V, López-Briz E, Carbonell Sanchis R, Gonzalvez Perales JL, Bort-Marti S. Megestrol acetate for treatment of anorexia-cachexia syndrome. Cochrane Database Syst Rev. 2013;2013(3):CD004310. 10.1002/14651858.CD004310.pub3. 10.1002/14651858.CD004310.pub3 PMC6418472 23543530

[47] Madeddu C, Macciò A, Panzone F, Tanca FM, Mantovani G. Medroxyprogesterone acetate in the management of cancer cachexia. Expert Opin Pharmacother. 2009;10(8):1359–66. 10.1517/14656560902960162. 19445562 10.1517/14656560902960162

[48] Loprinzi CL, Michalak JC, Schaid DJ, Mailliard JA, Athmann LM, Goldberg RM, et al. Phase III evaluation of four doses of megestrol acetate as therapy for patients with cancer anorexia and/or cachexia. J Clin Oncol. 1993;11(4):762–7. 10.1200/JCO.1993.11.4.762. 8478668 10.1200/JCO.1993.11.4.762

[49] Wright TJ, Dillon EL, Durham WJ, Chamberlain A, Randolph KM, Danesi C, et al. A randomized trial of adjunct testosterone for cancer-related muscle loss in men and women. J Cachexia Sarcopenia Muscle. 2018;9(3):482–96. 10.1002/jcsm.12295. 29654645 10.1002/jcsm.12295 PMC5989774

[50] Bai Y, Hu Y, Zhao Y, Yu X, Xu J, Hua Z, et al. Anamorelin for cancer anorexia-cachexia syndrome: a systematic review and meta-analysis. Support Care Cancer. 2017;25(5):1651–9. 10.1007/s00520-016-3560-0. 28074289 10.1007/s00520-016-3560-0

[51] Hardy J, Greer R, Huggett G, Kearney A, Gurgenci T, Good P. Phase IIb randomized, placebo-controlled, dose-escalating, double-blind study of cannabidiol oil for the relief of symptoms in advanced cancer (MedCan1-CBD). J Clin Oncol. 2023;41(7):1444–52. 10.1200/JCO.22.01632. 36409969 10.1200/JCO.22.01632

[52] Kardinal CG, Loprinzi CL, Schaid DJ, Hass AC, Dose AM, Athmann LM, et al. A controlled trial of cyproheptadine in cancer patients with anorexia and/or cachexia. Cancer. 1990;65(12):2657–62. 10.1002/1097-0142(19900615)65:12%3c2657::aid-cncr2820651210%3e3.0.co;2-s. 2187585 10.1002/1097-0142(19900615)65:12<2657::aid-cncr2820651210>3.0.co;2-s

[53] Loprinzi CL, Goldberg RM, Su JQ, Mailliard JA, Kuross SA, Maksymiuk AW, et al. Placebo-controlled trial of hydrazine sulfate in patients with newly diagnosed non-small-cell lung cancer. J Clin Oncol. 1994;12(6):1126–9. 10.1200/JCO.1994.12.6.1126. 8201374 10.1200/JCO.1994.12.6.1126

[54] Del Fabbro E, Dev R, Hui D, Palmer L, Bruera E. Effects of melatonin on appetite and other symptoms in patients with advanced cancer and cachexia: a double-blind placebo-controlled trial. J Clin Oncol. 2013;31(10):1271–6. 10.1200/JCO.2012.43.6766. 23439759 10.1200/JCO.2012.43.6766 PMC3607670

[55] Solheim TS, Fearon KC, Blum D, Kaasa S. Non-steroidal anti-inflammatory treatment in cancer cachexia: a systematic literature review. Acta Oncol. 2013;52(1):6–17. 10.3109/0284186X.2012.724536. 23020528 10.3109/0284186X.2012.724536

[56] Sandhya L, Devi Sreenivasan N, Goenka L, Dubashi B, Kayal S, Solaiappan M, et al. Randomized double-blind placebo-controlled study of olanzapine for chemotherapy-related anorexia in patients with locally advanced or metastatic gastric, hepatopancreaticobiliary, and lung cancer. J Clin Oncol. 2023;41(14):2617–27. 10.1200/JCO.22.01997. 36977285 10.1200/JCO.22.01997

[57] Yennurajalingam S, Willey JS, Palmer JL, Allo J, Del Fabbro E, Cohen EN, et al. The role of thalidomide and placebo for the treatment of cancer-related anorexia-cachexia symptoms: results of a double-blind placebo-controlled randomized study. J Palliat Med. 2012;15(10):1059–64. 10.1089/jpm.2012.0146. 22880820 10.1089/jpm.2012.0146 PMC3438834

[58] Jatoi A, Ritter HL, Dueck A, Nguyen PL, Nikcevich DA, Luyun RF, et al. A placebo-controlled, double-blind trial of infliximab for cancer-associated weight loss in elderly and/or poor performance non-small cell lung cancer patients (N01C9). Lung Cancer. 2010;68(2):234–9. 10.1016/j.lungcan.2009.06.020. 19665818 10.1016/j.lungcan.2009.06.020 PMC5951722

